# A single-dose intranasal immunization with a novel bat influenza A virus-vectored MERS vaccine provides effective protection against lethal MERS-CoV challenge

**DOI:** 10.1128/mbio.01107-25

**Published:** 2025-06-30

**Authors:** Lei Shi, Sawrab Roy, Yuekun Lang, Yuhan Wen, William J. Mitchell, Wenyu Yang, Liping Wang, Jialin Zhang, Heidi Liu, John P. Driver, Malik Peiris, Wenjun Ma

**Affiliations:** 1 Department of Veterinary Pathobiology, College of Veterinary Medicine, University of Missouri 219018, Columbia, Missouri, USA; 2 Department of Molecular Microbiology and Immunology, School of Medicine, University of Missouri 219013, Columbia, Missouri, USA; 3 MU Center for Influenza and Emerging Infectious Diseases, University of Missouri 14716, Columbia, Missouri, USA; 4 Division of Animal Sciences, University of Missouri 169008 , Columbia, Missouri, USA; 5 Veterinary Medical Diagnostic Laboratory, College of Veterinary Medicine, University of Missouri 365852, Columbia, Missouri, USA; 6 School of Public Health, Li Ka Shing Faculty of Medicine, The University of Hong Kong 90397, Hong Kong, China; Virginia Polytechnic Institute and State University, Blacksburg, Virginia, USA

**Keywords:** MERS-CoV, bat influenza vectored MERS vaccine, safety and immunogenicity, cross-protection

## Abstract

The threat to global health security posed by Middle East respiratory syndrome coronavirus (MERS-CoV) and emerging MERS-like coronaviruses highlights the need to develop safe and efficient vaccines. Viral vector vaccines have been shown to be effective and are widely used to prevent various viral diseases because they mimic natural infection and induce a more comprehensive immune response. Herein, we developed a novel bat influenza A virus-based vaccine vector by replacing the open reading frame of either bat influenza hemagglutinin or neuraminidase with that of the hemagglutinin-esterase-fusion gene from influenza D virus, which can infect multiple species, including humans and camels. We then generated a temperature-sensitive, cold-adapted, and attenuated MERS vaccine candidate expressing the clade A MERS-CoV spike S1, referred to as Len_S1, using the developed bat influenza vector and demonstrated its safety and immunogenicity. A single-dose intranasal immunization with Len_S1 protected human dipeptidyl-peptidase-4 (hDPP4) transgenic mice from a lethal MERS-CoV challenge. Notably, a two-dose immunization with Len_S1 completely blocked viral replication and lung damage in challenged mice. Further studies revealed that intranasal immunization with Len_S1 in mice elicited mucosal, humoral, and cellular immune responses. Moreover, sera collected from Len_S1-immunized mice were able to cross-neutralize multiple clades of MERS-CoVs. Collectively, these results indicate that Len_S1 is a safe and effective MERS vaccine that induces a comprehensive immune response and provides cross-protection against diverse clades of MERS-CoVs.

**IMPORTANCE:**

Middle East respiratory syndrome coronavirus (MERS-CoV) is an important zoonotic virus with pandemic potential that continues to evolve within dromedary camels. However, no licensed vaccine is currently available. Viral vector-based vaccines represent a promising platform, with demonstrated efficacy in preventing viral diseases. In this study, we developed a bat influenza virus-vectored MERS vaccine, Len_S1, that is safe and immunogenic. Intranasal immunization of human dipeptidyl-peptidase-4 (hDPP4)-transgenic mice with Len_S1 induced humoral, mucosal, and cellular immune responses and provided effective protection against a lethal MERS-CoV challenge. Importantly, sera collected from immunized mice cross-neutralized three distinct clades of MERS-CoVs. Our results indicate that Len_S1 is a promising vaccine candidate with the potential to prevent MERS-CoV infection and mitigate the risk of future epidemics and pandemics.

## INTRODUCTION

Middle East respiratory syndrome coronavirus (MERS-CoV) is a lethal zoonotic pathogen, sometimes causing major outbreaks within and beyond the Middle East. As of May 2024, 2,613 laboratory-confirmed human cases of MERS have been reported from 27 countries to the World Health Organization, including 943 associated deaths (36% mortality). Although MERS-CoV has not resulted in a large, sustained pandemic like severe acute respiratory syndrome coronavirus 2 (SARS-CoV-2), it continues to evolve within dromedary camels across a broad geographic region (the Middle East, North, West, and East Africa, and Central Asia), raising concerns about the potential for a future pandemic (1). Zoonotic infection has been associated with human-adaptive mutations (2). MERS-like coronaviruses have been identified in pangolins and lesser bamboo bats, and further studies have shown that they utilize the human dipeptidyl-peptidase-4 (hDPP4) receptor for cell entry, infect hDPP4 transgenic mice, and induce histological lung injury (3, 4). Additionally, MERS-like coronaviruses have been isolated from bat species in South Africa and several European countries (5). However, these viruses use the SARS-CoV-2 receptor, angiotensin-converting enzyme 2 (ACE2), for cell entry rather than hDDP4 (6, 7). This raises the possibility that newly emerging MERS-like coronaviruses utilizing ACE2 may possess evolutionary advantages in tropism and transmissibility, as exemplified by the highly transmissible SARS-CoV-2 Omicron variants (8, 9). Vaccines have been shown to control the COVID-19 pandemic induced by SARS-CoV-2. However, no commercial MERS vaccines are currently available. While COVID-19 vaccines were rapidly developed and intramuscularly administered to prevent disease, they are less effective at eliciting mucosal immunity and preventing transmission (10). Effective vaccines capable of eliciting mucosal immunity remain a critical need (11). Given that MERS-CoV continues to cause sporadic outbreaks with high mortality rates, the development of safe and effective vaccines is essential to prevent future epidemics and pandemics.

Influenza A virus (IAV) has become an attractive vaccine vector due to a well-established reverse genetics system (12), the ease with which viral genes can be modified (13), and established protocols for large-scale virus production (14). Although various vaccine candidates have been developed using the IAV-vectored platform (15–18), its limited gene capacity constrains its ability to deliver large antigens (13, 19–22). For example, efforts to develop IAV-vectored SARS-CoV-2 vaccines have only succeeded in incorporating the receptor-binding domain, not the full-length spike (S) or S1 subunit into recombinant IAVs (23, 24). One strategy to increase the gene capacity of IAVs is to flank the foreign gene with packaging signals of HA or NA segments (25). Previous studies have shown that replacing the HA and NA genes of IAV with the hemagglutinin-esterase-fusion (HEF) glycoprotein gene of influenza C virus (ICV) or the vesicular stomatitis virus glycoprotein (VSV-G) gene allows for the rescue of recombinant IAVs expressing green fluorescent protein (GFP) (25, 26). This strategy enables the generation of replication-competent IAVs that carry large foreign antigen genes as live virus vaccines. Several strategies have been employed to improve the safety of such live influenza vaccines and vectors, including truncation of the NS1 protein (27, 28) and generation of temperature-sensitive, cold-adapted strains (29, 30). However, the use of live influenza vaccines and IAV-vectored vaccines raises concerns about potential reassortment with circulating seasonal or endemic IAVs, which could result in novel viruses with increased pathogenicity and transmissibility (18).

The genomes of two novel IAV subtypes, H17N10 and H18N11, were discovered in bats in 2009–2010 (31, 32), and live bat influenza viruses were subsequently generated using reverse genetics (33, 34). *In vitro* studies have shown that only a limited number of cell lines are susceptible and permissive to these novel bat IAVs (35, 36), suggesting that many adaptive mutations are required for efficient replication (35). *In vivo*, neither clinical signs nor horizontal transmission were observed in bat IAV-infected mice and ferrets, despite transmission among bats—likely due to the very limited viral replication (34, 36). Poor infection and adaptation of bat IAVs in other species results in low pathogenicity and a lack of pre-existing immunity. Importantly, bat IAVs do not reassort with conventional IAVs due to incompatibilities in packaging signals among viral gene segments (37–39). These unique features make bat IAVs promising vaccine vectors. We and others have developed bat influenza-vectored swine and avian influenza vaccines and demonstrated their efficacy in relevant hosts (40–42). In this study, we developed a novel bat IAV-based vector by replacing the HA and NA segments of bat influenza with HEF from influenza D virus (IDV), which infects multiple species, including humans, camels, and pigs (43). Using this system, we generated bat influenza-vectored MERS vaccines expressing the MERS-CoV spike S1 subunit, which were further modified and attenuated to enhance S1 expression. We evaluated the safety, immunogenicity, and protective efficacy of the developed vaccine candidates in both standard inbred and hDPP4-transgenic mice.

## RESULTS

### Construction of a bat influenza A virus-based vector and MERS vaccine candidates expressing MERS-CoV spike S1

**Fig 1:**
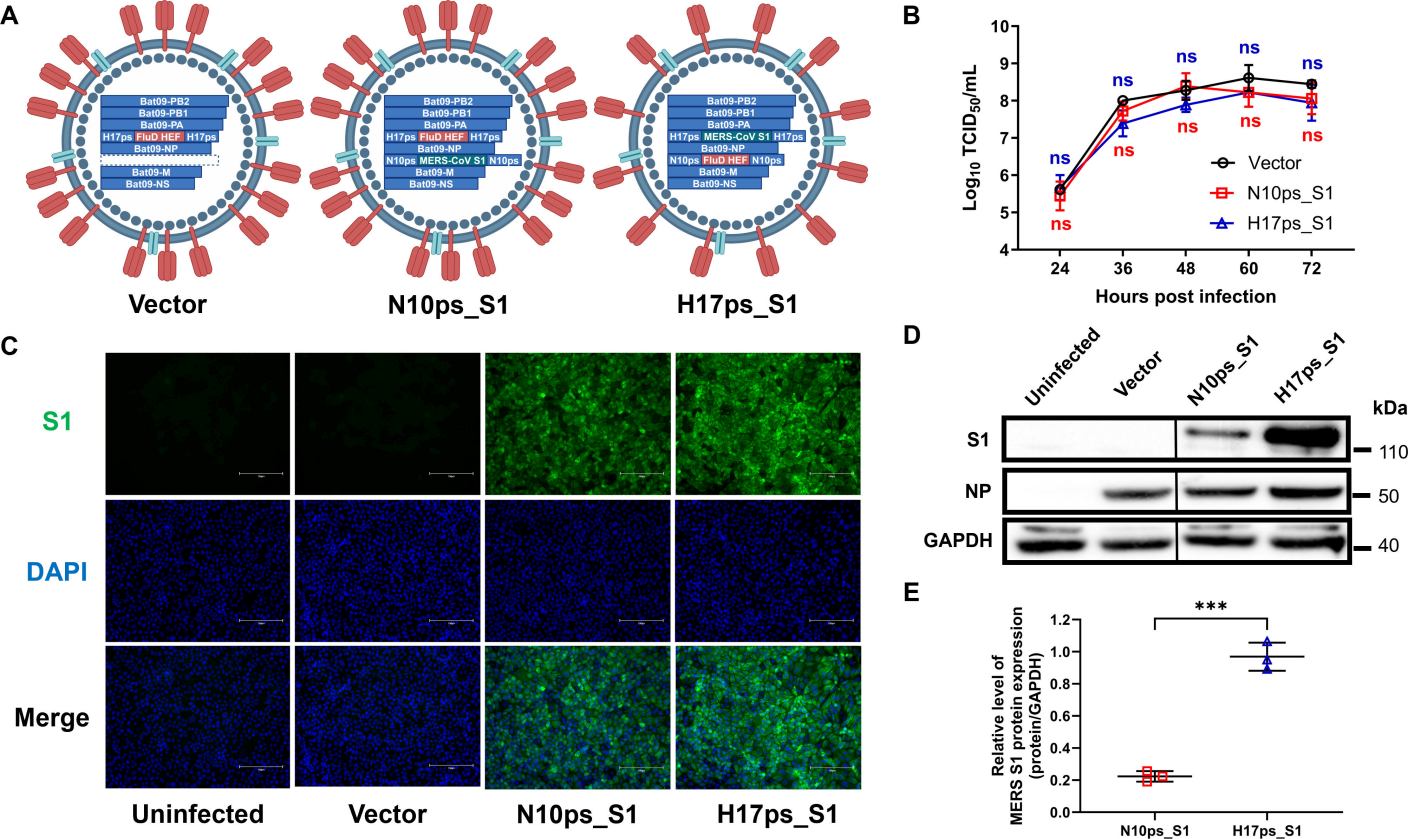
Construction and characterization of a bat influenza virus-based vector and potential MERS vaccine candidates expressing the spike S1 subunit. (**A**) Schematic diagrams of the construction of the bat influenza A virus-based vector and MERS vaccine candidates expressing the MERS-CoV spike S1 subunit. ORFs of influenza D virus HEF and MERS-CoV spike S1 were flanked with either Bat09 HA or NA packaging signals, as indicated. Recombinant viruses containing seven or eight segments were rescued and designated as vector, N10ps_S1, and H17ps_S1. (**B**) Madin-Darby canine kidney (MDCK) cells were infected with the indicated viruses at a multiplicity of infection (MOI) of 0.01. Supernatants were harvested at 24, 36, 48, 60, and 72 hours post-infection (hpi), and viral growth curves were determined. (**C**) MDCK cells were infected with the indicated viruses at an MOI of 0.1. At 24 hpi, cells were fixed and stained to detect MERS-CoV S1 expression. Nuclei were stained with DAPI. Scale bar: 150 µm. (**D**) MDCK cells were infected with the indicated virus at an MOI of 0.1, and cell lysates were collected at 24 hpi. Expression of MERS-CoV S1 and Bat09 NP was detected by western blotting. (**E**) MERS-CoV S1 expression in N10ps_S1- and H17ps_S1-infected cells was relatively quantified by normalizing to glyceraldehyde-3-phosphate dehydrogenase (GAPDH). The data represent results from three independent experiments.

Unlike conventional IAVs and IBVs, which require both surface HA and NA proteins to complete the viral life cycle (44), the HEF glycoprotein from either ICVs or IDVs can perform receptor binding, membrane fusion, and receptor cleaving functions and is sufficient to support viral replication in multiple mammalian cell types (45). To develop the bat influenza A virus-based vector, the HEF open reading frame (ORF) from an IDV (D/bovine/Kansas/14-22/2012) was flanked with the packaging signals of the HA gene (H17ps) from a bat H17N10 IAV (Bat09) (Fig. 1A). Along with the other six internal genes from Bat09, the viral vector containing seven segments was rescued via reverse genetics. To develop bat influenza-based MERS vaccine candidates, the MERS-CoV spike S1 ORF was flanked with the packaging signals of the NA gene (N10ps) from Bat09 (Fig. 1A). The HA-to-NA ratio in IAV particles ranges from 4:1 to 10:1, depending on the virus subtype and genetic background (46–48), and switching the packaging signals of the IAV HA and NA genes can enhance NA expression (49). To increase S1 expression, we switched the packaging signals flanking the HEF and S1 ORFs (Fig. 1A). All the start codons in the 3′ ends of both H17ps and N10ps were silenced by site-directed mutagenesis as described previously (37) to ensure HEF and S1 expression. Two recombinant viruses expressing S1 were rescued and referred to as N10ps_S1 and H17ps_S1, respectively.

Both N10ps_S1 and H17ps_S1 were purified through two rounds of plaque assay. The S1 genes in both recombinant viruses were stable after 10 serial passages, as confirmed by sequencing. Both N10ps_S1 and H17ps_S1 replicated efficiently and exhibited similar growth kinetics on Madin-Darby canine kidney (MDCK) cells when compared to the vector virus with seven segments (Fig. 1B). This indicated that the expression of a foreign gene and switching of packaging signals did not impair viral replication and growth. S1 expression was detected in the cytoplasm of MDCK cells infected with either N10ps_S1 or H17ps_S1 (Fig. 1C) and confirmed by western blotting (Fig. 1D). Notably, significantly higher S1 expression was observed in H17ps_S1-infected cells compared to N10ps_S1-infected cells (Fig. 1E). Because switching the packaging signals significantly increased S1 expression without compromising replication, H17ps_S1 was selected as the parental virus for further attenuation and characterization.

### Attenuation of bat influenza-vectored MERS vaccine candidates

**Fig 2:**
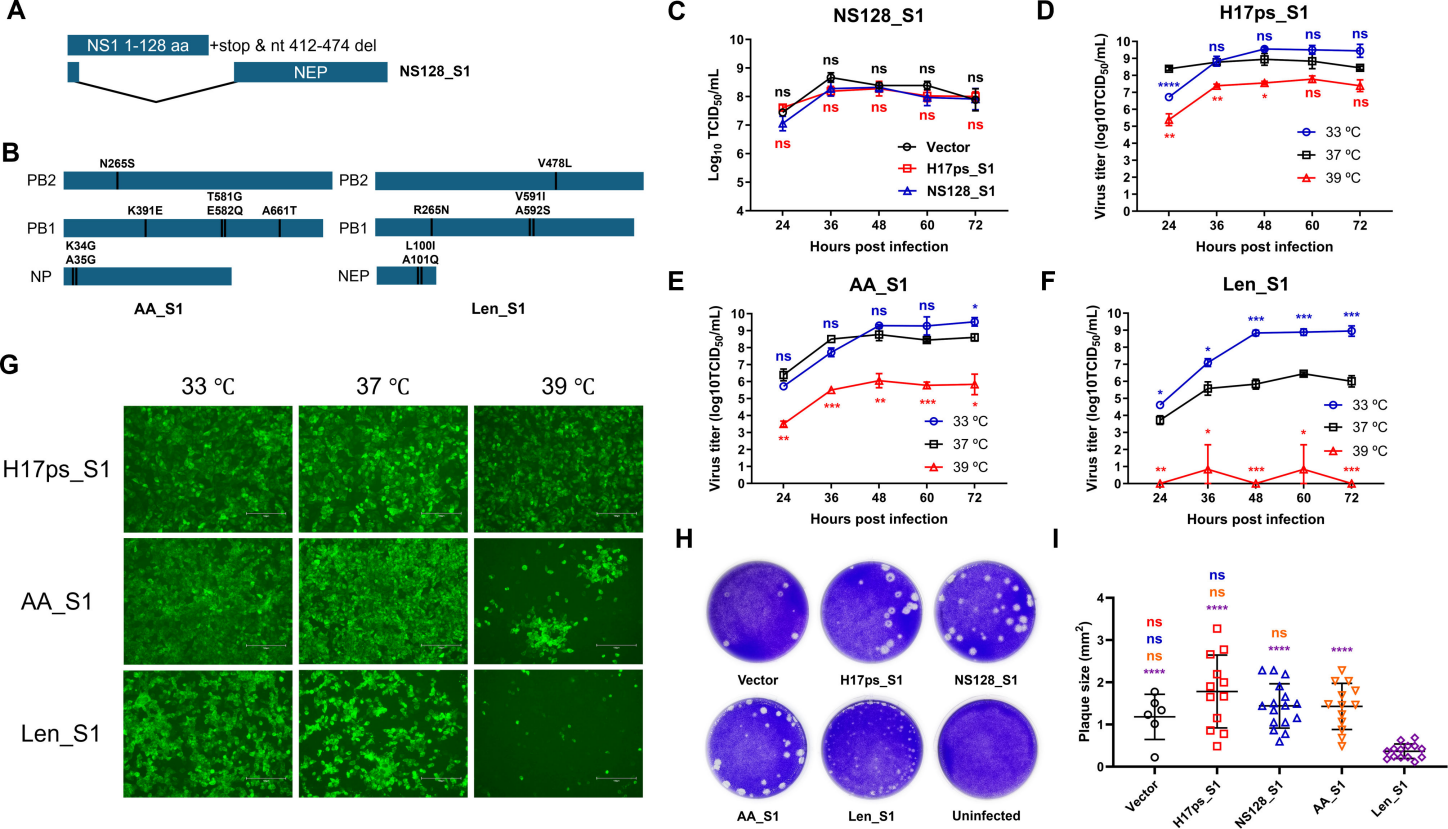
Attenuation of bat influenza-vectored MERS vaccine candidates. (**A, B**) Schematic diagrams of two approaches used to attenuate bat influenza-vectored MERS vaccine candidates. (**A**) The NS1 protein was truncated by introducing three consecutive stop codons at the 128th codon and deleting nucleotides 412-474. (**B**) Amino acids responsible for the *ts*, *ca*, and *att* phenotypes in AA and Len viruses were introduced into the corresponding genes of Bat09. (**C**) MDCK cells were infected with vector, H17ps_S1, or NS128_S1 at an MOI of 0.01 at 37°C. Supernatants were collected at 24, 36, 48, 60, and 72 hpi to compare viral growth dynamics. (**D–F**) MDCK cells were infected with H17ps_S1, AA_S1, or Len_S1 at an MOI of 0.01 and cultured at 33°C, 37°C, or 39°C. Supernatants were harvested at the indicated time points and titrated on MDCK cells at 37°C to determine the growth kinetics of parental H17ps_S1 (**D**), AA_S1 (**E**), and Len_S1 (**F**). (**G**) MDCK cells were infected with H17ps_S1, AA_S1, or Len_S1 at an MOI of 0.1 and cultured at 33°C, 37°C, or 39°C. At 24 hpi, cells were fixed and stained for S1 expression using an MERS-CoV S1 antibody. Nuclei were stained with DAPI. Scale bar: 150 µm. (**H**) MDCK cells were infected with 10-fold dilutions (10^−1^ to 10^−6^) of the vector or the indicated viruses. At 3 days post-infection (dpi), cells were fixed with cold methanol and stained with crystal violet. (**I**) Plaque sizes formed by each virus were quantified using ImageJ software.

To develop safer bat influenza-vectored MERS vaccines, we further attenuated the H17ps_S1 candidate by truncating the NS1 protein (40, 50–52) and generating temperature-sensitive (*ts*), cold-adapted (*ca*), and attenuated (*att*) strains (30, 53, 54). To truncate the NS1, the NS segment of the H17ps_S1 genome retained a truncated NS1 gene encoding amino acids 1-128 (Fig. 2A). The NS1-truncated recombinant virus, named NS128_S1, was rescued. NS128_S1 exhibited growth kinetics similar to those of parental H17ps_S1 (Fig. 2C), formed plaques of comparable size (Fig. 2H and I), and expressed S1 effectively as shown by immunofluorescence assay (IFA) (Fig. S1B).

Amino acids responsible for the *ts*, *ca,* and *att* phenotypes have been identified in human H2N2 pandemic viruses. For the US A/Ann Arbor/6/60 H2N2 IAV (AA) strain, these phenotypes are mapped to PB2 (N265S), PB1 (K391E, E581G, and A661T), and NP (D34G) (54). For the Russian A/Leningrad/134/17/57 H2N2 IAV (Len) strain, they are mapped to PB2 (V478L), PB1 (K265N and V591I), and NEP (M100I) (53). To generate an H17ps_S1-based *ts*, *ca,* and *att* strain, we compared the protein sequences of AA, Len, and Bat09. Most amino acids associated with these phenotypes were conserved in Bat09 (Fig. S1A), but two amino acid differences were identified at certain critical positions (Fig. S1A). Accordingly, we introduced single or double mutations to ascertain the underlying functional domains that confer the *ts*, *ca,* and *att* phenotypes (Fig. 2B). Two recombinant viruses with respective amino acid substitutions were rescued and named AA_S1 and Len_S1. To determine whether AA_S1 and Len_S1 acquired the *ts*, *ca*, and *att* phenotypes, we evaluated their growth kinetics in MDCK cells at 33°C, 37°C, and 39°C, using parental H17ps_S1 as the control. Both H17ps_S1 and AA_S1 showed similar replication at 33°C and 37°C but exhibited significantly reduced titers at 39°C (Fig. 2D and E). In contrast, Len_S1 showed markedly reduced replication at 37°C and nearly complete loss of replication at 39°C (Fig. 2F). Plaque size (Fig. 2H and I) and cytopathic effects (CPEs) at 37°C were also significantly reduced in Len_S1-infected cells (Fig. S1C), while no substantial changes were observed in H17ps_S1- or AA_S1-infected cells. IFA results confirmed S1 expression in Len_S1- and AA_S1-infected cells (Fig. 2G). Consistent with replication data, S1 expression in H17ps_S1-infected cells was only mildly affected by temperature, whereas expression in AA_S1- and Len_S1-infected cells was markedly reduced at 39°C, indicating enhanced temperature sensitivity. Taken together, these results demonstrate that introducing Len strain-specific mutations into the H17ps_S1 genome confers *ts*, *ca*, and *att* phenotypes.

### Len_S1 is safe and immunogenic in mice

**Fig 3:**
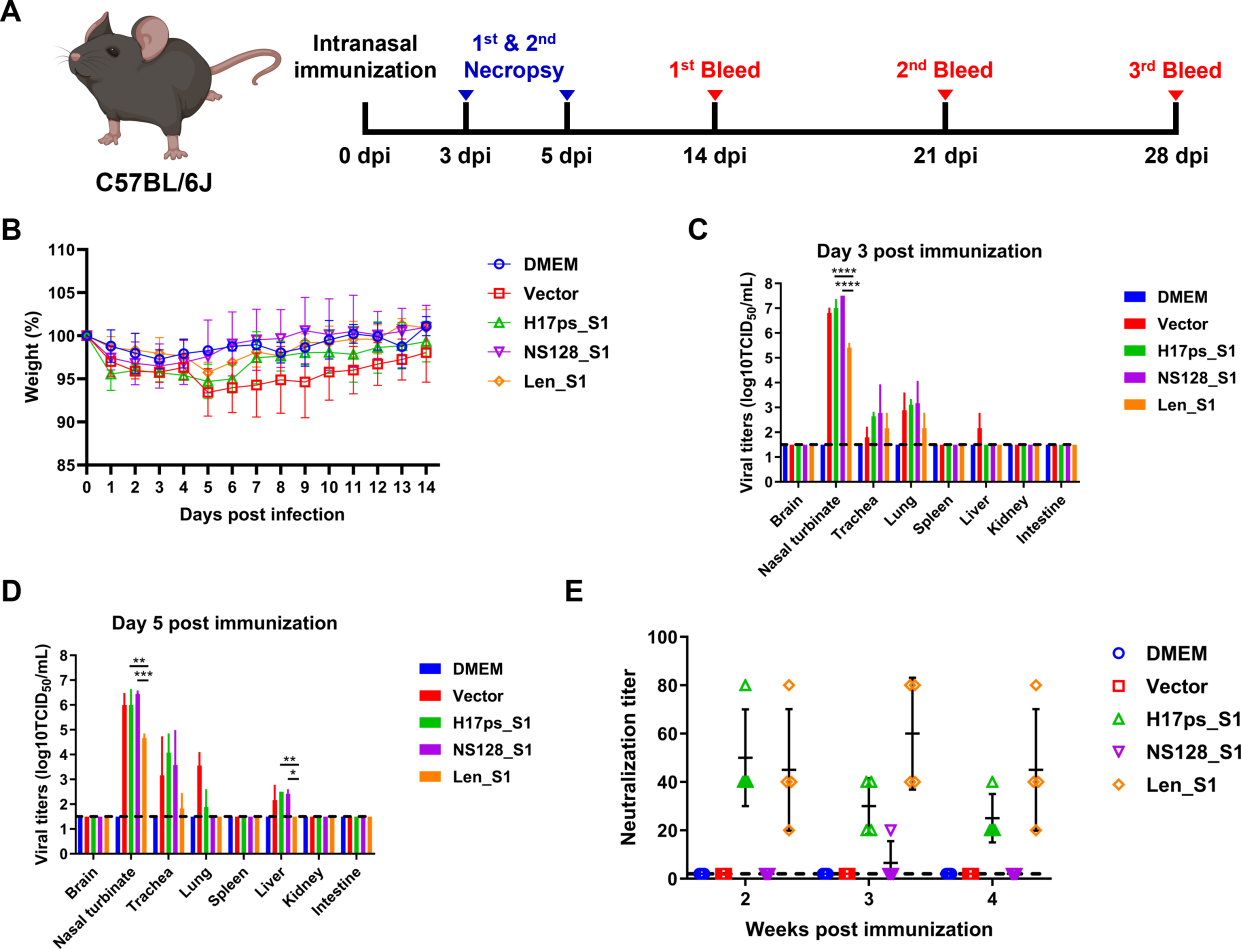
Len_S1 demonstrates safety and immunogenicity in mice. (**A**) Schematic overview of the experimental design to evaluate the safety and immunogenicity of MERS vaccine candidates in C57BL/6J mice. (**B**) Weight change over 14 days in mice intranasally mock-immunized with DMEM or immunized with 10^6^ TCID_50_ of the vector, H17ps_S1, NS128_S1, or Len_S1. (**C, D**) Virus replication in organ tissues of immunized mice. Three mice per group were necropsied at 3 and 5 dpi, and the indicated tissues were collected, homogenized, and analyzed for viral titers on MDCK cells. The dashed line indicates the limit of detection (10^1.5^ TCID_50_/mL). (**E**) Detection of neutralizing antibodies in sera from mice immunized with the indicated viruses. Serum samples were collected at 2, 3, and 4 weeks post-immunization, serially diluted, and incubated with rVSV-eGFP-S to determine neutralizing antibody titers using Vero E6 cells.

To evaluate the safety and immunogenicity of the vaccine candidates, we intranasally immunized groups of C57BL/6J mice with a single dose (10^6^ TCID_50_) of H17ps_S1, NS128_S1, or Len_S1. Groups of mice immunized with Dulbecco’s Modified Eagle’s medium (DMEM) or the vector virus served as controls. No obvious clinical signs were observed in mice in any group during a 14-day observation period. Compared to DMEM-immunized mice, both H17ps_S1- and vector-immunized mice lost approximately 5% of their body weight between 5 and 6 days post-infection (dpi), while Len_S1-immunized mice experienced only minor weight loss followed by rapid recovery (Fig. 3B). In all four groups, the virus was predominantly detected in the upper respiratory tract (nasal turbinates and trachea) at 3 and 5 dpi, and in the lungs at 3 dpi. At 5 dpi, the virus was also detected in the lungs of vector- or H17ps_S1-immunized mice but not in those immunized with Len_S1 or NS128 (Fig. 3C and D). Notably, the virus was detected in the livers of mice immunized with H17ps_S1, NS128-S1, and the vector at 5 dpi, but not in those immunized with Len_S1. Significantly lower viral loads were observed in the nasal turbinates of Len_S1-infected mice compared to those immunized with the other two vaccine candidates. No significant differences in virus titers were detected in the trachea or lungs at 3 and 5 dpi among the vaccine groups. These findings indicate that Len_S1 exhibits an attenuated phenotype *in vivo* and is likely safer than the other two vaccine candidates and the parental vector.

To determine the immunogenicity of the vaccine candidates, we performed the neutralization assay on serum samples collected from mice using a recombinant vesicular stomatitis virus (VSV) expressing GFP and the MERS-CoV spike (rVSV-eGFP-S), in which the VSV glycoprotein was replaced with the MERS-CoV spike. The results showed that all H17ps_S1- and Len_S1-infected mice seroconverted at 2 weeks post-immunization (wpi), and neutralizing antibodies (NAbs) were detectable at 4 wpi (Fig. 3E). No significant difference in NAb titers was observed between these two groups. Surprisingly, NS128_S1-infected mice did not show seroconversion at 2 wpi, despite efficient replication in the mouse upper respiratory tract. Only one out of four remaining NS128_S1-infected mice seroconverted at 3 wpi. However, the NAbs were not detected at 4 wpi, suggesting that NS1 truncation may reduce the immunogenicity of H17ps_S1. Taken together, both H17ps_S1 and Len_S1 were immunogenic and induced durable NAb responses in mice. Compared to the parental virus H17ps_S1, Len_S1 demonstrated improved safety without compromising immunogenicity.

### A single-dose intranasal immunization with Len_S1 elicits a comprehensive immune response in hDPP4 mice

**Fig 4:**
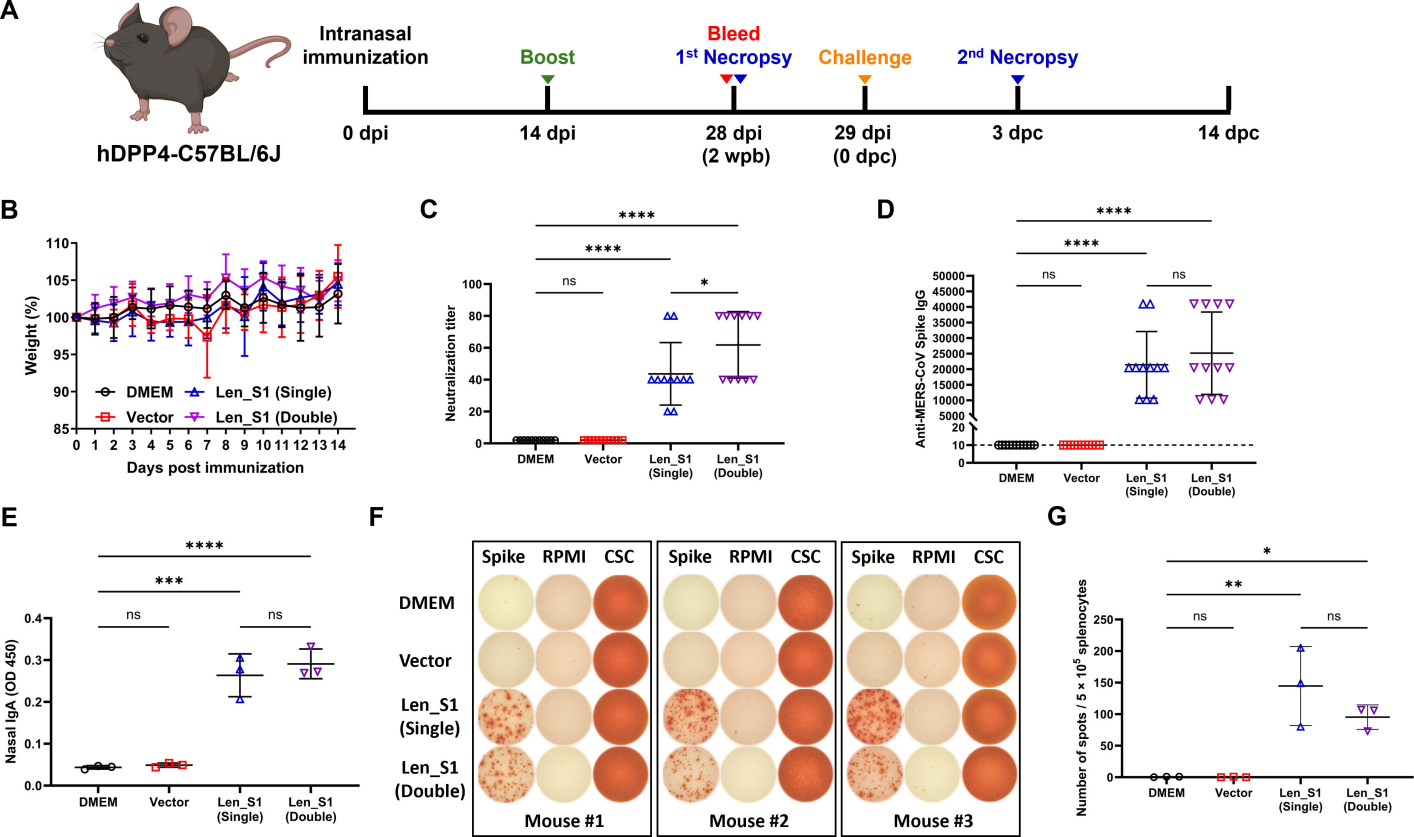
A single-dose intranasal immunization with Len_S1 elicits a comprehensive immune response in mice. (**A**) Experimental design to evaluate immune responses and efficacy of Len_S1 in hDPP4-transgenic C57BL/6J mice. (**B**) Body weight changes of mice immunized with the vector or Len_S1, monitored over 14 days post-immunization. (**C**) Neutralizing antibody titers induced by one or two doses of Len_S1. Sera were collected at 4 wpi, serially diluted, and incubated with rVSV-eGFP-S to determine neutralizing titers. (**D**) Serum IgG levels in Len_S1-immunized mice. Sera collected at 4 wpi were analyzed for MERS-CoV spike-specific IgG titers by ELISA. The dashed line indicates the detection limit (10-fold dilution). (**E**) Nasal IgA levels in Len_S1-immunized mice. Nasal turbinates were collected from three euthanized mice at 4 wpi, and nasal washes were assessed for spike-specific IgA titers by ELISA. (**F**) T cell responses in Len_S1-immunized mice. Spleens were collected from three mice per group at 4 wpi. Splenocytes (5 × 10^5^ cells) were stimulated with 5 µg/mL purified MERS-CoV spike trimers, and spike-specific responses were measured by ELISPOT assay. (**G**) Number of spot-forming units per well was quantified using ImmunoSpot software.

Next, we intranasally immunized transgenic C57BL/6J mice expressing hDPP4 (hDPP4 mice) (55) with either a single or two doses of Len_S1 to evaluate vaccine-induced immune responses. Vector- and vehicle (DMEM)-immunized mice were used as controls (Fig. 4A). Consistent with our results in standard C57BL/6J mice, hDPP4 mice immunized with either vector or Len_S1 did not exhibit weight loss or clinical signs during the 14-day observation period (Fig. 4B). Furthermore, Len_S1 induced 100% seroconversion and generated NAb levels at 28 dpi that were comparable to those observed in standard C57BL/6J mice (Fig. 4C). We also measured MERS-CoV spike-specific IgG antibodies in mouse serum using an enzyme-linked immunosorbent assay (ELISA) method. High concentrations of spike-specific IgG were detected in both singly and doubly immunized mice prior to MERS-CoV challenge (Fig. 4D). Interestingly, boosting significantly increased NAb titers but did not alter spike-specific IgG levels. As the upper respiratory mucosa is the first line of defense against MERS-CoV infection, we measured the spike-specific IgA antibody titers in the nasal tissues of mice to assess mucosal immunity. As expected, IgA antibodies were detected in all Len_S1-immunized mice but not in mice from either the control or vector groups at 2 weeks post-booster (Fig. 4E). To assess the MERS-CoV spike-specific cellular response, splenocytes from different groups were stimulated with purified MERS-CoV spike trimers. Enzyme-Linked Immunospot assay (ELISPOT) analysis revealed a strong T-cell response against the MERS-CoV spike in all Len_S1-immunized mice (Fig. 4F and G). Notably, the booster did not significantly enhance mucosal or T-cell responses. Taken together, a single-dose intranasal immunization with Len_S1 is sufficient to elicit a comprehensive immune response in hDPP4 mice.

### A single-dose intranasal immunization with Len_S1 is sufficient to protect against a lethal MERS-CoV challenge

**Fig 5:**
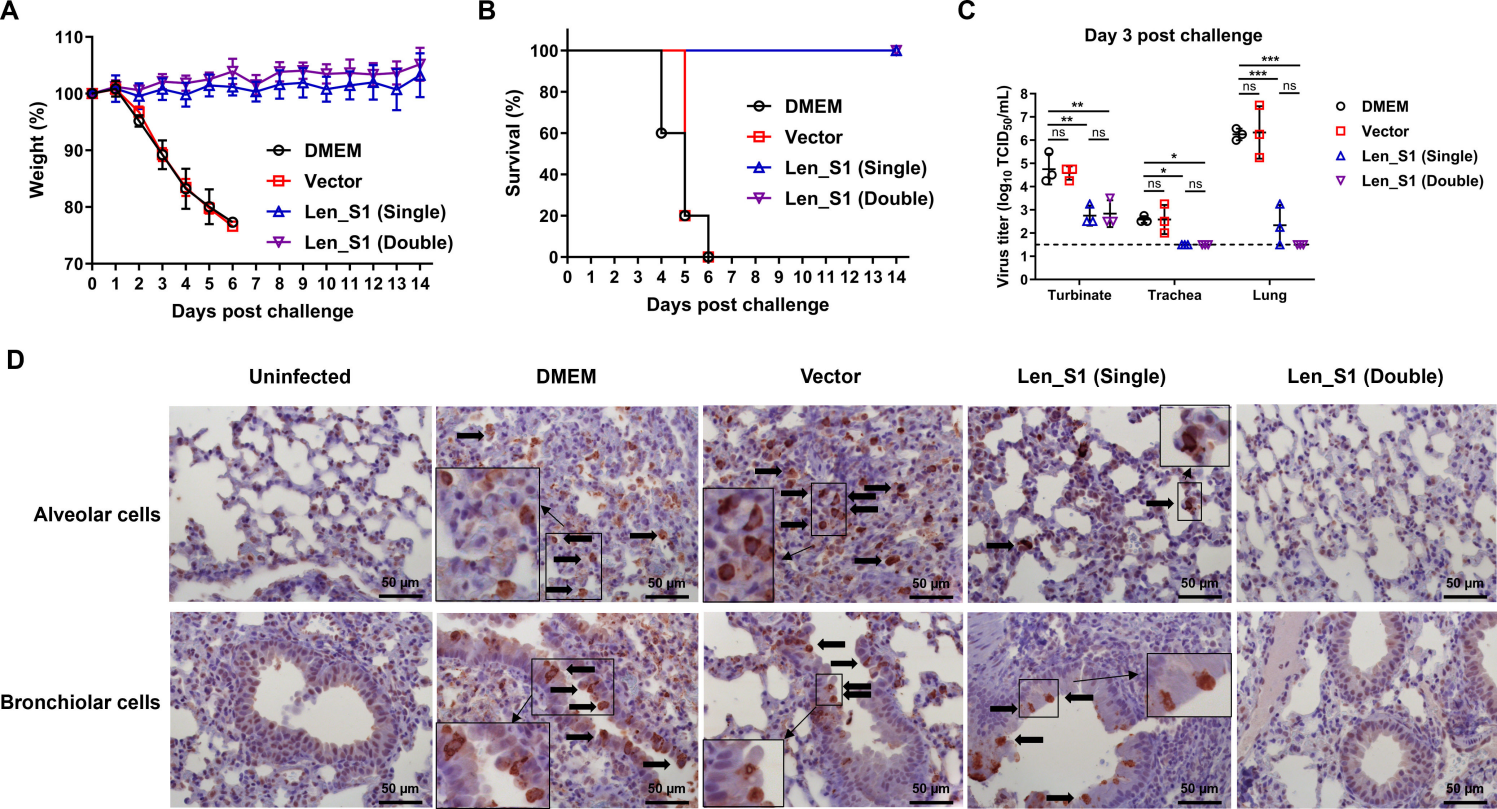
A single-dose intranasal immunization with Len_S1 provides effective protection against lethal MERS-CoV challenge. Groups of hDPP4-transgenic C57BL/6J mice were intranasally challenged with 10-fold LD_50_ of the mouse-adapted MERS-CoV Clone 6.1.2 at 4 weeks post-immunization with the vector or Len_S1. Clinical signs and body weight were monitored daily. Three mice per group were necropsied for the collection of nasal turbinates, trachea, and lungs for viral titration and histopathological analysis. (**A**) Body weight of mice in each group over 14 days post-challenge. (**B**) Survival percentage of mice in each group over 14 days post-challenge. (**C**) Viral titers in the nasal turbinates, trachea, and lungs of three mice per group. Titers were determined using Vero 81 cells. Dashed line indicates the limit of detection (10^1.5^ TCID_50_/mL). (**D**) Detection of MERS-CoV N antigen in mouse lung tissues. Lungs were fixed, and N antigen was visualized by immunohistochemistry. Arrows indicate N antigen-positive alveolar and bronchiolar cells. Scale bar: 50 µm.

To evaluate the efficacy of Len_S1, we challenged singly or doubly immunized hDPP4 mice with a 10-fold 50% lethal dose (LD_50_) of a mouse-adapted MERS-CoV strain (55, 56) at 28 dpi (Fig. 4A). Following the challenge, all mice in the mock- or vector-immunized groups progressively lost weight (Fig. 5A) and exhibited severe clinical signs, including labored breathing, trembling, and ruffled fur. In contrast, mice immunized with either a single or two doses of Len_S1 showed no weight loss or clinical signs over the 14 days post-challenge (dpc) (Fig. 5A). By 6 dpc, all mice in the DMEM and vector groups succumbed to infection. No difference in disease severity was observed between these two groups (Fig. 5B), indicating that the vector did not provide protection against MERS-CoV. Virus titers were significantly higher in the respiratory tract tissues (nasal turbinates, trachea, and lungs) of mock- and vector-immunized mice at 3 dpc compared to those in mice immunized with one or two doses of Len_S1 (Fig. 5C). In Len_S1-immunized mice, low MERS-CoV titers were primarily detected in the nasal turbinates (Fig. 5C). No virus was detected in the trachea or lung tissues of double-immunized mice. In contrast, a virus was detected in the lungs of two out of three singly immunized mice, although none showed viral presence in the trachea (Fig. 5C).

Histopathological analysis revealed that both mock- and vector-immunized mice exhibited severe inflammation in the alveolar spaces and interalveolar septae, with multifocal necrosis of bronchiolar epithelial cells (Fig. S2). In comparison, Len_S1-immunized mice displayed markedly reduced inflammation, especially in those that received two doses, which showed far fewer macrophages, neutrophils, and fibrin deposits (Fig. S2). Consistent with viral titer data, MERS-CoV N antigen was detected in alveolar and bronchiolar cells in mock, vector, and singly immunized mice (Fig. 5D). However, the number of N antigen-labeled cells was significantly lower in the singly immunized group, suggesting that Len_S1 reduced virus replication in the lungs. In contrast, no N antigen-positive alveolar or bronchiolar cells were observed in uninfected controls or in mice that received two doses of Len_S1 (Fig. 5D). These results demonstrate that a single-dose intranasal immunization with Len_S1 is sufficient to protect against lethal MERS-CoV challenge, preventing both morbidity and mortality. However, boosting is required to achieve effective immunity that completely prevents lung inflammation and pneumonia, in addition to morbidity and mortality.

### Len_S1 induces cross-neutralizing antibodies

Pooled sera from mice vaccinated with single or double doses of Len_S1 and subsequently challenged were tested against different clades of MERS-CoVs, including clade A EMC, clade B camel/Al-Hasa-KFU-HKU13/2013 (AH13), and clade C camel/Nigeria/NV1657/2016 (NV1657), using the PRNT_90_ assay (Table 1). Pooled sera collected prior to virus challenge showed neutralizing activity against EMC (clade A), with a PRNT_90_ titer of 80, and against both AH13 (clade B) and NV1657 (clade C) MERS-CoVs, with PRNT_90_ titers of 40 and 20–40, respectively (Table 1). Interestingly, no significant differences in antibody titers were observed between mice that received one or two vaccinations. Moreover, mice immunized with one or two doses of Len_S1 produced increased neutralizing antibody titers against all three MERS-CoV clades after challenge. A fourfold to eightfold increase in antibody titers was observed in singly immunized mice, whereas a twofold increase was observed in doubly immunized mice (Table 1). These findings indicate that Len_S1 induces cross-reactive neutralizing antibodies against multiple clades of MERS-CoVs.

**TABLE 1:** Cross-reactivity of pooled sera from immunized and/or challenged mice against different MERS-CoV clades

Samples	PRNT_90_ titers		
	EMC (clade A)	AH13 (clade B)	NV1657 (clade C)
Vector (2 weeks post-immunization)	<10	<10	<10
Len_S1 (single) (2 weeks post-immunization)	80	40	20
Len_S1 (double) (2 weeks post-immunization)	80	40	40
Vector (4 weeks post-immunization)	<10	<10	<10
Len_S1 (single) (4 weeks post-immunization)	80	40	40
Len_S1 (double) (4 weeks post-immunization)	80	40	40
Len_S1 (single) (2 weeks post-challenge)	≥320	≥320	160
Len_S1 (double) (2 weeks post-challenge)	160	80	80

## DISCUSSION

Several MERS vaccines have undergone phase 1 trials, including a DNA vaccine (57) and adenovirus- and vaccinia virus-vectored vaccines (58, 59). Compared to DNA vaccines, vectored vaccines are considered more effective at eliciting protective immune responses, particularly in mucosal tissues. However, pre-existing immunity has been shown to interfere with orthopoxvirus- (60–62) and adenovirus-vectored vaccines (63, 64). Therefore, clinical data will be required to demonstrate the efficacy of vectored MERS vaccines in individuals with pre-existing immunity for this platform to be successful. IAV-vectored vaccines have shown promise due to their ability to elicit comprehensive immune responses (17). Nevertheless, significant drawbacks remain, including the potential for reassortment with circulating IAVs and limited capacity to deliver large antigen genes. To address these issues, the current study explored the development of a MERS vaccine using a novel bat influenza virus vector for the following reasons: (i) novel bat influenza viruses do not reassort with conventional IAVs (37–39), which addresses safety concerns; (ii) we replaced both the bat influenza HA and NA genes with the HEF gene from IDV, which performs the combined functions of HA and NA and is more stable than HA proteins in IAV/IBV (65). This substitution enables the bat influenza vector to tolerate and deliver large antigens by flanking them with HA or NA packaging signals; and (iii) most species, including humans, do not have pre-existing immunity to novel bat IAVs (33). Additionally, to enhance S1 expression, we switched the bat influenza HA and NA packaging signals to flank the HEF and S1 ORFs. We found that S1 expression was significantly enhanced when the S1 ORF was flanked by the Bat09 HA packaging signals compared to when it was flanked by NA packaging signals, consistent with the known HA/NA ratio in influenza virus particles (46, 47, 66). Furthermore, this construct stably expressed S1 after serial passaging, indicating its promise as a vaccine platform.

To improve safety, we employed two approaches to attenuate the bat influenza-vectored MERS vaccines. Interestingly, NS1 truncation did not attenuate virus replication, contrary to previous reports (40). This may be due to differences in parental virus strains. We successfully generated a *ts*, *ca*, and *att* MERS vaccine strain by introducing key amino acid substitutions identified in the H2N2 A/Leningrad/134/17/57 strain (53), rather than those from the A/Ann Arbor/6/60 strain (30). The successful attenuation using conventional IAV strategies suggests that bat IAVs may share more characteristics with conventional IAVs than previously expected.

We demonstrated that our MERS vaccine candidate, Len_S1, was attenuated and genetically stable. Importantly, Len_S1 was shown to be safe in standard and hDPP4-transgenic C57BL/6J mice, as evidenced by the absence of weight loss or clinical signs. Serological evidence suggests that, in addition to swine and cattle, IDVs can infect a broad range of hosts, including camelids and humans (43, 67). Moreover, IDV has been detected in respiratory and/or gastrointestinal tissues of infected mice and guinea pigs (68, 69). In contrast, Len_S1 was detected only in the respiratory tract of mice, predominantly in the upper respiratory tract, consistent with its *ts*, *ca*, and *att* phenotypes, further supporting its safety profile. Our bat influenza-based vector and MERS vaccine candidates exhibit a natural tropism for the respiratory tract and are likely applicable to multiple species. These findings suggest potential utility in delivering antigens from other respiratory pathogens, in addition to MERS-CoV, across different hosts.

Our study shows that mice immunized with one or two doses of Len_S1 are protected against a lethal MERS-CoV challenge, as evidenced by the absence of morbidity, mortality, and clinical signs, including weight loss. Two doses of Len_S1 provided superior protection compared to a single dose, as no virus or histopathological evidence of disease was detected in the lungs of doubly immunized mice. Both single-dose and two-dose immunization induced robust cellular and humoral immune responses, including neutralizing antibodies, mucosal IgA, and MERS-CoV spike-specific T cell responses. Both NAbs and T cell responses likely contribute to the observed protection. Despite relatively low serum neutralizing antibody titers, high MERS-CoV spike-specific IgG levels were observed in mice immunized with single or two doses of Len_S1, suggesting that many vaccine-induced S1-specific antibodies are non-neutralizing. Prior studies have shown that non-neutralizing antibodies can confer effective protection *in vivo* via Fc-mediated mechanisms (70–73), such as antibody-dependent cellular cytotoxicity and antibody-dependent cellular phagocytosis. Further studies on the role of these non-neutralizing antibodies could yield important insights into the mechanisms of protection conferred by our vaccine. Boosting with Len_S1 increased serum IgG and nasal IgA levels compared to a single dose, although these increases were not statistically significant. Notably, neutralization antibody titers were significantly elevated in boosted mice. These enhanced humoral responses may contribute to the improved protection observed in doubly vaccinated mice. Furthermore, our findings suggest that Len_S1 may be suitable for vaccination in animals with preexisting immunity.

The MERS-CoV phylogeny comprises three major clades: clades A, B, and C. Clades A and B viruses are associated with dromedary camels in the Arabian Peninsula, while clade C viruses are associated with camels in Africa (74). Predicting which clade or strain will cause future outbreaks remains difficult. To assess whether our vaccine candidate can provide cross-clade protection, we found that sera from mice immunized with single or double doses of Len_S1 cross-neutralized clades B and C viruses, in addition to the homologous clade A strain. This indicates that Len_S1 may provide cross-protection against diverse MERS-CoV clades. Although Len_S1 provides effective protection against a lethal homologous MERS-CoV challenge and induces cross-neutralizing antibodies, its ability to prevent MERS-CoV transmission remains unclear and warrants further investigation.

In conclusion, we have developed a novel bat IAV-vectored MERS vaccine and demonstrated its safety and immunogenicity. Both single- and two-dose intranasal immunizations conferred effective protection against lethal MERS-CoV challenge by eliciting robust humoral and cellular immune responses. Additionally, antibodies induced by Len_S1 cross-neutralized all three major MERS-CoV clades. These findings highlight Len_S1 as a promising vaccine candidate with the potential to provide broad protection. Further studies are needed to assess efficacy under different vaccination regimens (e.g., in combination with adjuvanted inactivated vaccines), in animals with IDV or MERS-CoV antibodies, and in other susceptible species such as camelids and non-human primates.

## MATERIALS AND METHODS

### Cell lines, viruses, and animals

MDCK cells were cultured in minimum essential medium (MEM) supplemented with 10% fetal bovine serum (FBS) and 1% antibiotic-antimycotic solution. HEK293T cells were cultured in Opti-MEM medium supplemented with 10% FBS and 1% antibiotic-antimycotic solution. Huh-7 and Vero 81 cells, kindly provided by Dr. Stanley Perlman at the University of Iowa, and BSR-T7/5 cells, kindly provided by Dr. Karl-Klaus Conzelmann at Ludwig Maximilians-University Munich, were cultured in DMEM supplemented with 10% FBS and 1% antibiotic-antimycotic solution.

Wild-type human/EMC/2012 (GenBank ID: JX869059.2) and mouse-adapted MERS-CoV strains (55) were kindly provided by Dr. Stanley Perlman at the University of Iowa. The clade B camel/Al-Hasa-KFU-HKU13/2013 (AH13) (GenBank ID: KJ650295.1) and the clade C camel/Nigeria/NV1657/2016 (NV1657) (GenBank ID: MG923475.1) viruses were stored at the University of Hong Kong. The VSV reverse genetics system was kindly provided by Dr. Sean Whelan at Washington University in St. Louis. The modified vaccinia Ankara virus expressing T7 polymerase (MVA-T7), used in recombinant VSV rescue, was kindly provided by Dr. Bernard Moss at the National Institutes of Health.

Standard C57BL/6J mice were purchased from the Jackson Laboratory. Transgenic C57BL/6J mice expressing hDPP4 under the control of human keratin 18 promoter were kindly provided by Dr. Stanley Perlman at the University of Iowa and propagated at the University of Missouri.

### Generation and characterization of bat influenza virus vector and vectored MERS vaccine candidates

The PB2, PB1, PA, NP, M, and NS segments of the H17N10 A/little yellow-shouldered bat/Guatemala/153/2009 (Bat09) virus, the EMC/2012 MERS-CoV S1 ORF flanked with the packaging signals of Bat09 HA or NA, and the HEF ORF from D/bovine/Kansas/14-22/2012 flanked with the packaging signals of the Bat09 HA or NA segment were cloned into the bidirectional transcription plasmid pHW2000 (12). Recombinant viruses were rescued by co-transfecting seven or eight plasmids into 293T cells, and the resulting supernatants were passaged on MDCK cells. Briefly, seven or eight plasmids (500 ng each) were added to 200 µL of Opti-MEM. Then, 12 µL of X-tremeGENE HP DNA Transfection Reagent (Sigma, 6366236001) was added and gently mixed with the plasmids. The mixture was incubated at room temperature for 20 minutes before being applied to confluent 293T monolayers. At 3 days post-transfection, the 293T cells were subjected to two freeze-thaw cycles, and the collected supernatant was passaged onto MDCK cells incubated in MEM supplemented with 0.3% BSA and 1 µg/mL of tosyl phenylalanyl chloromethyl ketone (TPCK)-treated trypsin until CPEs were observed. All rescued viruses were purified by two rounds of the plaque assay and subsequently passaged ten times. The viral genomes were confirmed by sequencing.

To generate an NS1-truncated recombinant virus, the NS segment was modified by inserting three stop codons at the 128th codon of the NS1, as described previously (37), and cloned into the pHW2000 plasmid. To generate recombinant viruses with *ts*, *ca*, and *att* phenotypes, the protein sequences of internal genes from Bat09, A/Ann Arbor/6/60 H2N2 (AA), and A/Leningrad/134/17/57 H2N2 (Len) strains were aligned and compared using MEGA software. Amino acids in PB2, PB1, NP, and NEP associated with the *ts*, *ca*, and *att* phenotypes in the AA or Len strains (30, 53) were introduced into the corresponding Bat09 genes via site-directed mutagenesis (Fisher, A14604). Recombinant viruses were rescued and confirmed by sequencing. To determine viral growth kinetics, MDCK cells were infected with each virus at a multiplicity of infection (MOI) of 0.01 and incubated at 33°C, 37°C, and/or 39°C. The supernatants were collected at 24, 36, 48, 60, and 72 hours post-infection, and virus titers were determined on MDCK cells and calculated using the Reed–Muench method.

### Western blotting and immunofluorescence assays

A confluent monolayer of MDCK cells on 6-well plates was infected with each indicated virus at an MOI of 0.1. At 24 hours post-infection, infected cells were harvested and lysed using RIPA Lysis and Extraction Buffer (Fisher, 89900) supplemented with 1× Halt Protease Inhibitor Cocktail (Fisher, 78429). After SDS-PAGE, proteins were transferred to a PVDF membrane. S1 expression was detected using an anti-MERS-CoV S1 rabbit polyclonal antibody (1:1,000, Sino Biological, 40069-T52), and viral NP was detected using an anti-influenza A NP rabbit polyclonal antibody (1:1,000, Fisher, PA5-32242). A horseradish peroxidase (HRP)-conjugated goat anti-rabbit IgG (H+L) (1:2,000, Fisher, A16096) was used as the secondary antibody to detect both S1 and NP. GAPDH was detected using a mouse monoclonal GAPDH antibody (1:1,000, Santa Cruz, sc-166545) and an HRP-conjugated goat anti-mouse IgG (H+L) (1:2,000, Fisher, A16066) as the secondary antibody. S1 expression in virus-infected cells was relatively quantified by normalizing grayscale intensity to GAPDH using ImageJ software. For IFA, infected cells were fixed at 24 hours post-infection with cold methanol and blocked with 5% skim milk. S1 protein was detected using the anti-MERS-CoV S1 rabbit polyclonal antibody (1:1,000, Sino Biological, 40069-T52) and an Alexa Fluor 488 goat anti-rabbit IgG (H+L) cross-adsorbed secondary antibody (1:1,000, Fisher, A-11008). Cell nuclei were stained with 4′,6-diamidino-2-phenylindole, dihydrochloride (DAPI) (1:5,000, Fisher, 62247).

### Plaque assay

Virus stocks were serially diluted from 10^−1^ to 10^−9^ in MEM infection medium containing 0.3% BSA, 1% antibiotic-antimycotic solution, and 1 µg/mL of TPCK-treated trypsin. MDCK cells in 6-well plates were infected with the diluted virus and incubated for 1 hour at 37°C. After removing the supernatant, 2 mL of an agarose overlay, which was prepared by mixing 1.6% low-gelling-temperature agarose (Lonza, 50110) and 2× MEM in a 1:1 ratio and supplemented with 2 µg/mL TPCK-treated trypsin, was added to each well. After solidifying at room temperature, the plate was incubated upside-down at 37°C for 2–3 days until translucent plaques appeared. Single plaques were picked into MEM for purification and stored at −80°C for future amplification. To analyze plaque sizes, cold methanol was added to fix the cells, followed by crystal violet staining. The plaque size was measured using ImageJ software.

### Mouse studies

To determine the safety and immunogenicity of the vaccine candidates, fifty 7–8-week-old female C57BL/6J mice (10 mice per group) were divided into five groups. Each mouse was intranasally inoculated with 50 µL of either DMEM (control) or 10^6^ TCID_50_ of a vaccine candidate under isoflurane anesthesia. Body weight and clinical symptoms were monitored daily for 14 days. On days 3 and 5 post-immunization, three mice from each group were necropsied, and the remaining four mice were maintained for 4 weeks post-immunization. Tissues including brain, nasal turbinate, trachea, lung, spleen, liver, kidney, and intestine were collected from necropsied animals to assess virus replication. Blood samples were collected at 2, 3, and 4 weeks post-immunization, and serum was isolated to determine neutralizing antibody titers.

Forty-four male and female transgenic hDPP4 mice, aged 12–15 weeks, were divided into four groups (11 mice per group) to evaluate vaccine efficacy and immune responses. Each mouse was intranasally mock-immunized or immunized with 10^6^ TCID_50_ of either the vector or Len_S1. Body weight and clinical symptoms were monitored daily for 14 days post-immunization. One group of Len_S1-immunized mice received a booster dose via the same route at 14 days post-immunization. At 4 weeks post-immunization, blood was collected to assess humoral immune responses. In parallel, three randomly selected mice from each group were sacrificed, and nasal turbinates and spleens were collected to evaluate mucosal and cellular immune responses. The remaining mice in each group were intranasally mock-challenged or challenged with 50 µL of DMEM containing a 10-fold 50% lethal dose (LD_50_) of mouse-adapted MERS-CoV Clone 6.1.2. Body weight and clinical symptoms were monitored daily for 14 days post-challenge. On day 3 post-challenge, three randomly selected mice from each group were necropsied, and respiratory tissues, including nasal turbinates, trachea, and lungs, were collected for viral titration and histopathological analysis. Half of each tissue was used for virus quantification, and the other half was fixed in buffered 10% formalin for histopathological analysis.

Tissue homogenates were prepared in prefilled tubes containing beads (Precellys, P000911-LYSK0-A) using a ratio of 1 mL of DMEM per 0.1 g of tissue, supplemented with 2× antibiotic-antimycotic solution. Supernatants were collected following centrifugation. Supernatant from intestinal homogenates was additionally filtered through 0.22 µm filters to minimize contamination. Virus titers from homogenates were determined using MDCK (for vaccine strains) or Vero 81 cells (for MERS-CoV) and calculated using the Reed–Muench method.

### Histopathology and immunohistochemistry

Mouse lung tissues collected at 3 dpc were fixed in 10% buffered formaldehyde for at least 14 days prior to removal from the LIDR BSL-3 laboratory. For H&E staining, lung sections from MERS-CoV-infected and uninfected control mice were processed using standard procedures to evaluate the pathological lesions. Alveolar and bronchiolar regions were assessed for each animal. For immunohistochemistry, lung sections from infected and uninfected mice were deparaffinized and subjected to a polymer-based immunohistochemistry protocol (Dako) to detect viral antigens. Prior to applying the primary antibody, sections were blocked with hydrogen peroxide for 20 minutes, washed, and then further blocked with Sniper reagent (Biocare) for 15 minutes. The primary anti-MERS-CoV N rabbit polyclonal antibody (Fisher, PA5-119579) was applied at a 1:100 dilution for 30 minutes. In parallel control assays, lung sections from uninfected mice were incubated with the anti-MERS-CoV N antibody, while sections from infected mice were incubated with a rabbit IgG isotype antibody (Fisher 02-6102) at the same dilution as controls. A polymer-anti-rabbit Ab-enzyme complex (Envision-Dako) was used as the secondary antibody and incubated for 30 minutes. After washing in citrate buffer for 3 minutes, chromagen (Romulen red-Biocare) was added for 10 minutes. Sections were then examined under a microscope by a pathologist.

### Microneutralization assay and plaque reduction neutralization test

Recombinant VSV expressing eGFP and the MERS-CoV Spike (rVSV-eGFP-S), in which MERS-CoV S from the human/EMC/2012 replaced the VSV G in the genome of rVSV-eGFP-S, was rescued via the T7 promoter-based reverse genetics on BSR-T7/5 cells, as described previously (75, 76). The rescued virus was amplified and titrated on Vero E6 cells. Mouse sera were heat-inactivated at 56°C for 30 minutes and serially diluted. Diluted sera were incubated with 100 TCID_50_ of rVSV-eGFP-S at 37°C for 1 hour. The mixture was then transferred to confluent Vero E6 monolayers in 96-well plates and incubated at 37°C for 3 days. Viral replication was monitored daily via fluorescence microscopy. The neutralizing antibody titer was defined as the highest serum dilution that completely inhibited the eGFP signal. Non-neutralized virus produced large syncytia with strong eGFP signals.

MERS-CoVs from clade A (human/EMC/2012), clade B (AH13), and clade C (NV1657) were utilized to assess cross-neutralization by pooled sera from Len_S1-immunized hDPP4 mice. Neutralizing activity was determined using a plaque reduction neutralization test (PRNT) as described previously (77). Briefly, pooled sera were heat-inactivated at 56°C for 30 minutes, then twofold serially diluted and mixed with each MERS-CoV strain for 1 hour at 37°C. The virus-serum mixtures were added to confluent Vero cell monolayers and incubated for 1 hour at 37°C in a 5% CO_2_ incubator. After removal of the supernatant, cells were overlaid with 1% agarose (Lonza, 50110) in MEM supplemented with 2% FBS. Cells were fixed and stained after 3 days. Antibody titers were defined as the highest serum dilution that resulted in ≥90% (PRNT_90_) plaque reduction. All PRNT assays were performed in 24-well plates in duplicate for each serum sample.

### ELISA assay

To determine mucosal IgA and serum IgG levels, a standard ELISA protocol was performed. Briefly, purified MERS-CoV spike glycoprotein (BEI, NR-56131) was diluted to 1 µg/mL in 50 mM sodium bicarbonate buffer (pH 9.6) and used to coat 96-well ELISA plates overnight at 4°C. Plates were then blocked with 5% skim milk at 37°C for 2 hours, followed by three washes with PBS containing 0.5‰ of Tween-20 (PBST). Nasal wash samples and serially diluted serum samples (1:10 to 81,920 in 5% skim milk) were added to the plates. After 1 hour of incubation at 37°C, plates were washed six times with PBST. HRP-conjugated Goat anti-Mouse IgG (H+L) secondary antibody (1:2,000, Fisher, A16066) or HRP-conjugated Goat anti-Mouse IgA secondary antibody (1:2,000, Fisher, 62-6720), which was diluted in 5% skim milk, were added to the plates. After a further 1 hour incubation at 37°C, the plates were washed six times, and 100 µL per well of TMB substrate (Fisher, 34028) was added. After 15 minutes of incubation at room temperature, 100 µL per well of ELISA stop solution (Fisher, SS04) was added. Absorbance at 450 nm was measured using a FLUOstar Omega microplate reader (BMG LABTECH, type 0415). The cut-off value for defining a positive sample was calculated as 2 × (mean OD value of negative controls + standard deviation).

### ELISPOT assay

Splenocytes from immunized mice were isolated by gently grinding and passing spleen tissue through a 70 µm strainer (Falcon, 352350), then frozen in FBS containing 10% DMSO at −80°C. ELISPOT plates (Sigma, MSIPS4W10) were coated with 5 µg/mL PBS-diluted mouse IFN-γ capture antibody (BD, 51-2525KZ) and incubated at 4°C overnight. Plates were then blocked with RPMI 1640 supplemented with 10% FBS. Splenocytes were recovered, and 5 × 10^5^ cells per well were added to plates and stimulated with 5 µg/mL purified MERS-CoV spike glycoprotein for 36–48 hours. RPMI 1640 medium alone was used as the negative control, and a cell stimulation cocktail (Fisher, 00-4970-93) served as the positive control. After washing, IFN-γ expression was detected using a mouse IFN-γ detection antibody (BD, 51-1818KA) and AEC substrate (BD, 551951). Spot-forming units were enumerated using ImmunoSpot software.

### Statistical analysis

Growth curves of the rescued recombinant viruses were analyzed using two-way analysis of variance (ANOVA). Other data were analyzed using Student’s *t*-test or one-way ANOVA, followed by Tukey’s multiple comparison test. All *in vitro* data were representative of at least three independent experiments, and values were presented as mean ± SD. Data analysis was performed using GraphPad Prism 10 (GraphPad software). Significance levels were defined as follows: ****P < 0.0001; ***P < 0.001; **P < 0.01; *P < 0.05; ns: not significant, *P* > 0.05.

## Data Availability

Data generated and presented in this study are available upon request. Materials are available upon request through the filing of material transfer agreements.

## SUPPLEMENTAL MATERIAL

Figure S1 mbio.01107-25-s0001.tif Attenuation of bat influenza-vectored MERS vaccine candidates.

Figure S2 mbio.01107-25-s0002.tif H&E staining of lung tissues from challenged mice.

Legends mbio.01107-25-s0003.docx Legends for supplemental figures.

ASM does not own the copyrights to Supplemental Material that may be linked to, or accessed through, an article. The authors have granted ASM a non-exclusive, world-wide license to publish the Supplemental Material files. Please contact the corresponding author directly for reuse.
