# Supplementary figures and images for: A single-dose intranasal immunization with a novel bat influenza A virus-vectored MERS vaccine provides effective protection against lethal MERS-CoV challenge
Source: mBio. 2025 Jun 30;16(8):e01107-25. doi: 10.1128/mbio.01107-25 (PMC12345275; doi:10.1128/mbio.01107-25)

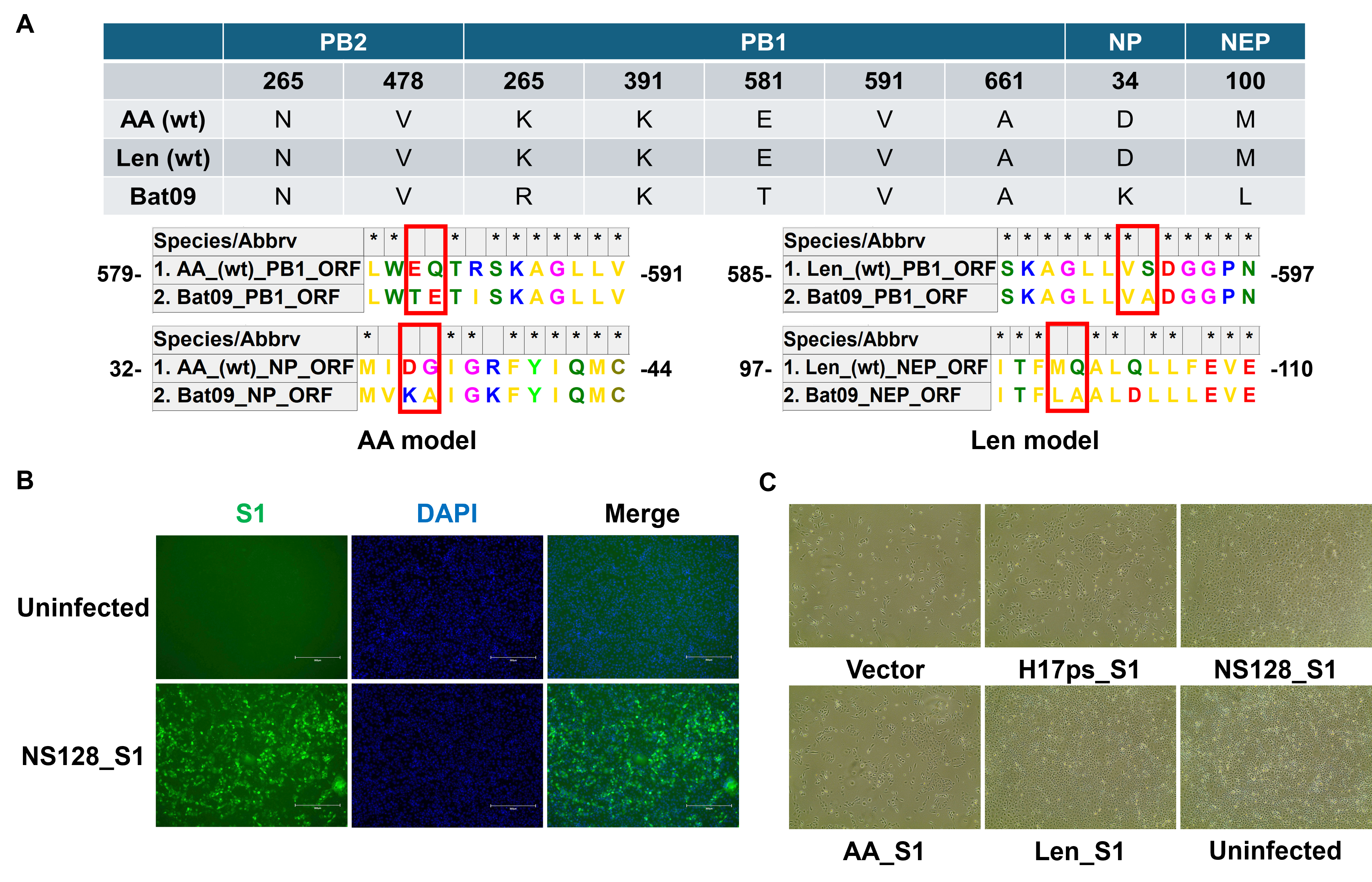

Supplement: Figure S1 — Attenuation of bat influenza-vectored MERS vaccine candidates. [file mbio.01107-25-s0001.tif]

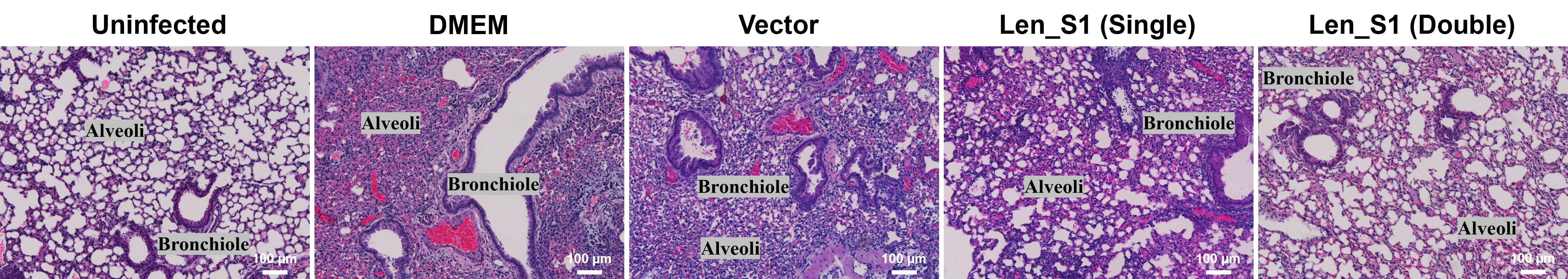

Supplement: Figure S2 — H&E staining of lung tissues from challenged mice. [file mbio.01107-25-s0002.tif]
