# Supplementary material for: A single-dose intranasal immunization with a novel bat influenza A virus-vectored MERS vaccine provides effective protection against lethal MERS-CoV challenge
Source: mBio. 2025 Jun 30;16(8):e01107-25. doi: 10.1128/mbio.01107-25 (PMC12345275; doi:10.1128/mbio.01107-25)
Supplement: Legends — for supplemental figures. [file mbio.01107-25-s0003.docx]

**Fig. S1. Attenuation of bat influenza-vectored MERS vaccine candidates.**

(A) Comparison of amino acid residues in PB2, PB1, NP, and NEP genes of the AA and Len H2N2 strains responsible for temperature-sensitive (*ts*), cold-adapted (*ca*)*,* and attenuated (*att*) phenotypes with those in the corresponding genes of Bat09. Red rectangles indicate the positions where double mutations were introduced into the respective Bat09 genes. (B) S1 expression in NS128_S1-infected MDCK cells. Cells were infected at an MOI of 0.1, fixed at 24 hpi, and stained by immunofluorescence assay (IFA) using a specific anti-MERS S1 antibody. Nuclei were stained with DAPI. Scale bar: 300 µm. (C) Cytopathic effect (CPE) induced by the indicated viruses in MDCK cells. Cells were infected with the vector or other indicated viruses at an MOI of 0.1, and CPE was observed at 48 hpi.

**Fig. S2. H&E staining of lung tissues from challenged mice.**

Lung tissues from mice collected at 3 dpc from each indicated group were stained with hematoxylin and eosin. The alveolar and bronchiolar regions were examined. Lung tissues from uninfected mice were used as controls, and no inflammation was observed in their alveoli or bronchioles. In contrast, lung sections from DMEM- and vector-immunized mice showed severe inflammation characterized by infiltration of macrophages and neutrophils, fibrin accumulation in alveolar spaces and interalveolar septae, and multifocal necrosis of epithelial cells in bronchioles. Mice immunized with a single dose of Len_S1 exhibited markedly reduced lung pathology, while those immunized with two doses of Len_S1 showed minimal or no inflammation. Scale bar: 100 µm.
